# Five-Year Comparative Study of Zygomatic and Subperiosteal Implants: Clinical Outcomes, Complications, and Treatment Strategies for Severe Maxillary Atrophy

**DOI:** 10.3390/jcm14030661

**Published:** 2025-01-21

**Authors:** Rafal Zielinski, Jakub Okulski, Martyna Piechaczek, Jan Łoś, Jerzy Sowiński, Monika Sadowska-Sowińska, Agata Kołkowska, Wojciech Simka, Marcin Kozakiewicz

**Affiliations:** 1 StomatologianaKsiezymMlynie, 16D Tymienieckiego, 90-365 Lodz, Poland; martyna.piechaczek2001@gmail.com; 2 Department of Maxillofacial Surgery, Medical University of Lodz, 113st Zeromskiego, 90-001 Lodz, Poland; jakub.okulski@gmail.com (J.O.); marcin.kozakiewicz@umed.lodz.pl (M.K.); 3 Miladent Private Dental Clinic, 80-283 Gdańsk, Poland; jan.janlos.o@gmail.com; 4 Private Dental Clinic, Tetmajera 3A Rd, 05-080 Izabelin C, Poland; jersow@gmail.com (J.S.); msadowska.gdansk@gmail.com (M.S.-S.); 5 Department of Inorganic Chemistry, Analytical Chemistry and Electrochemistry, Faculty of Chemistry, Silesian University of Technology, Krzywoustego St. 6, 44-100 Gliwice, Poland; agatkol653@student.polsl.pl (A.K.); wojciech.simka@polsl.pl (W.S.)

**Keywords:** zygomatic implants, subperiosteal implants, maxillary atrophy

## Abstract

**Background/Objectives**: Severe maxillary atrophy presents challenges in maxillofacial rehabilitation. This study compares the clinical outcomes of zygomatic and subperiosteal implants, focusing on implant survival, soft tissue management, and postoperative complications over a five-year follow-up. **Methods**: A retrospective cohort study analyzed 150 patients divided into two groups based on the type of implant. Zygomatic implants were assessed for immediate functional loading, procedural efficiency, and complications such as sinus-related issues and orbital damage. Subperiosteal implants were evaluated for their customized design, keratinized mucosa integration, and adaptation to severe anatomical limitations. Statistical analyses, including Chi-square tests, were used to determine significant differences (*p* < 0.05). **Results**: This study demonstrated differences in complication rates (sinus-related complications: 12.4% for zygomatic implants; peri-implantitis: 5.6% for subperiosteal implants). Implant survival rates were comparable (zygomatic: 96.3%, subperiosteal: 97.1%, *p* = 0.278). Zygomatic implants demonstrated higher incidences of sinus-related complications (12.4%) and risks of orbital damage. Subperiosteal implants exhibited superior soft tissue stability with fewer cases of peri-implantitis (5.6%, *p* < 0.05). Procedural duration was shorter for zygomatic implants (177 min vs. 123 min); however, subperiosteal implants allowed for re-implantation after failure, providing flexibility that was unavailable with zygomatic implants. **Conclusions**: Zygomatic implants excel in immediate functional loading and reduced procedural time but require advanced surgical expertise to mitigate anatomical risks. Subperiosteal implants offer a safer, customizable solution, particularly in anatomically complex cases. These findings emphasize the importance of individualized treatment planning and technological advancements in implant design to optimize clinical outcomes for patients with severe maxillary atrophy.

## 1. Introduction

The rehabilitation of patients with severe maxillary atrophy remains a significant challenge in maxillofacial surgery. Traditional bone grafting techniques, guided bone regeneration, and sinus augmentation have provided viable solutions but often involve lengthy treatment times, increased patient morbidity, and high financial costs [1,2]. Consequently, alternative approaches like zygomatic and subperiosteal implants have emerged as promising solutions for patients with insufficient bone volume.

Zygomatic implants, introduced as a solution for atrophic maxillae, are anchored in the zygomatic bone, bypassing the need for extensive bone grafting procedures [3,4]. Their unique design allows for immediate functional loading and reduced treatment time. The Zygomatic Anatomy-Guided Approach (ZAGA) and the ORIS criteria have become widely accepted classification systems, aiding in preoperative planning and assessing implant success rates [5]. However, concerns such as maxillary sinus penetration and orbital floor perforation remain significant risks [6].

Subperiosteal implants, initially developed in the mid-20th century, have experienced a technological revival with modern advancements in digital imaging and 3D printing. These implants rest directly on the bone under the periosteum and are secured with fixation screws, making them suitable for patients with severe bone atrophy where endosseous implants are not feasible [7,8]. Current innovations include safe, well-examined, custom-designed implants produced with laser sintering, improving implant fit and reducing intraoperative complications [9,10].

The clinical success of both implant types has prompted ongoing comparative research. While zygomatic implants offer immediate loading capabilities and less extensive surgery in terms of grafting, subperiosteal implants provide an individualized approach for severely atrophic maxillae without the risk of sinus involvement [6,7]. Evaluating factors such as implant stability, soft tissue management, patient-specific anatomy, and surgical complexity is critical in choosing the appropriate treatment modality [11].

While conventional implant surgical strategies, such as sinus lifts and guided bone regeneration, offer viable solutions for patients with severe maxillary atrophy, they are often associated with prolonged treatment timelines and increased patient morbidity. This study aims to address the clinical gap by providing a long-term comparative analysis of zygomatic and subperiosteal implants, offering insights into their respective advantages, limitations, and safety profiles.

## 2. Materials and Methods

### 2.1. Study Design

This retrospective cohort study aimed to compare clinical outcomes between patients treated with subperiosteal implants and those treated with zygomatic implants for the rehabilitation of severe maxillary atrophy. This study was conducted at a single dental implant clinic and included patients treated between 2010 and 2023. All patients were followed for a minimum of five years postoperatively.

The primary outcome measures included the following:

- Implant survival rates: Defined as removal of the implant.
- Prosthetic success: Stability and functionality of the prosthesis.
- Complications: Including implant exposure, recurrent swelling, peri-implant soft tissue health, and occurrence of sinus complications (e.g., sinusitis or mucosal thickening).

This study was approved by an ethical review board, and all participants provided informed consent prior to inclusion.

### 2.2. Statistical Analysis

Statgraphics Centurion version 18.1.12 (StatPoint Technologies, Warrenton, VA, USA) was used for statistical analyses. Statistical tests were Mann–Whitney U, Kruskal–Wallis, Fisher, and ANOVA. The detected relationships were assumed to be statistically significant when *p* < 0.05.

### 2.3. Patient Selection

Patients included in this study were required to meet several criteria to ensure the validity and relevance of the results. Eligible participants were aged between 35 and 80 years and had been diagnosed with severe maxillary atrophy classified as Cawood V or VI. All participants had undergone treatment with either subperiosteal or zygomatic implants with screw-retained bridge rehabilitation. Complete treatment records, including preoperative and postoperative CBCT imaging, were mandatory for inclusion, and patients were required to have a minimum follow-up period of five years post-surgery. Additionally, good systemic health was a prerequisite, defined as the absence of any uncontrolled medical conditions that could potentially affect implant outcomes.

Exclusion criteria were also strictly applied to maintain the integrity of the study. Active smokers or those who had not ceased smoking for at least six weeks prior to surgery were excluded, as were patients with uncontrolled diabetes mellitus (HbA1c > 7.5%) or other systemic diseases known to affect bone metabolism. Individuals with a history of head or neck radiation therapy were also excluded due to the potential impact on implant success. Furthermore, patients with incomplete follow-up data or those who were non-compliant with follow-up appointments were not considered for the study. In the subperiosteal implant study group, simultaneous tooth extraction and subperiosteal implant placement in the same alveolar process were also criteria for exclusion. However, in the zygomatic implant group, tooth extractions were performed alongside implant placement in some patients.

These criteria ensured a homogenous and reliable patient cohort for evaluating the outcomes of subperiosteal and zygomatic implants.

### 2.4. Patient Cohorts

The study population was divided into distinct groups based on the type and configuration of the implants used in their treatment (Table 1).

In the subperiosteal implant group, 89 patients were categorized into three subgroups according to the implant configurations:

Group 1: 51 patients treated with two individual subperiosteal implants, each featuring three multiunits.

Group 2: 24 patients who received two individual subperiosteal implants combined with one or two conventional implants, with each subperiosteal implant featuring two multiunits.

Group 3: 14 patients treated with a single subperiosteal implant featuring two, three, or four multiunits.

In the zygomatic implant group, 81 patients were divided into four subgroups based on the number and placement of implants:

Group 1: 14 patients treated with four zygomatic implants combined with one or two conventional implants.

Group 2: 15 patients treated with four zygomatic implants exclusively.

Group 3: 46 patients treated with two zygomatic implants combined with four conventional implants.

Group 4: 6 patients treated with three zygomatic implants combined with one or two conventional implants.

This detailed grouping allowed for a nuanced analysis of implant configurations and their outcomes. Each group within both the subperiosteal and zygomatic implant cohorts had specific characteristics and treatment protocols, providing a robust basis for comparative evaluation.

To ensure homogeneity, all surgeries were performed by a single experienced operator trained in both subperiosteal and zygomatic implant placement techniques. Preoperative CBCT imaging was used for planning and to confirm the severity of maxillary atrophy, ensuring consistency across patient selection.

### 2.5. Surgical Procedures

#### 2.5.1. Subperiosteal Implants

Subperiosteal implants were custom designed using advanced digital workflows. Preoperative CBCT imaging provided detailed anatomical data for designing implants tailored to each patient’s maxillary morphology. In all patients, implants were used on edentulous maxilla. In cases where tooth extractions were performed simultaneously with the placement of individual implants, the rami of the implants did not extend to encompass the post-extraction socket. These implants were fabricated using direct metal laser sintering (DMLS) technology from medical-grade titanium alloy.

##### Preoperative Preparation

All patients received prophylactic antibiotics (1 g Penicillin 24 h preoperatively, then every 12 h for seven days). Surgical procedures were performed under either general anesthesia with nasal intubation or local anesthesia using articaine with epinephrine, depending on patient and surgical complexity.

##### Surgical Technique

A crestal incision was made, extending bilaterally from one maxillary tuberosity to the other. A full-thickness palatal flap was then carefully elevated to expose the alveolar ridge and the infrazygomatic crest, providing access to the underlying bone structures. Using a reverse Langebeck retractor, the surgeon meticulously accessed the zygomatic bone while preserving the infraorbital nerve to prevent neural complications.

**Figure 1:**
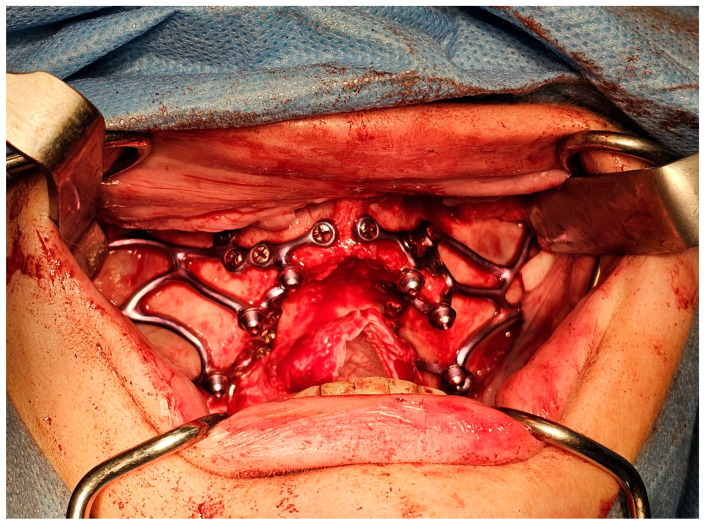
MaI Implant^®^ (Integra Implants^®^, Lodz, Poland) placement secured with self-tapping screws. The photo was taken before the implant was covered with autologous bone chips.

**Figure 2:**
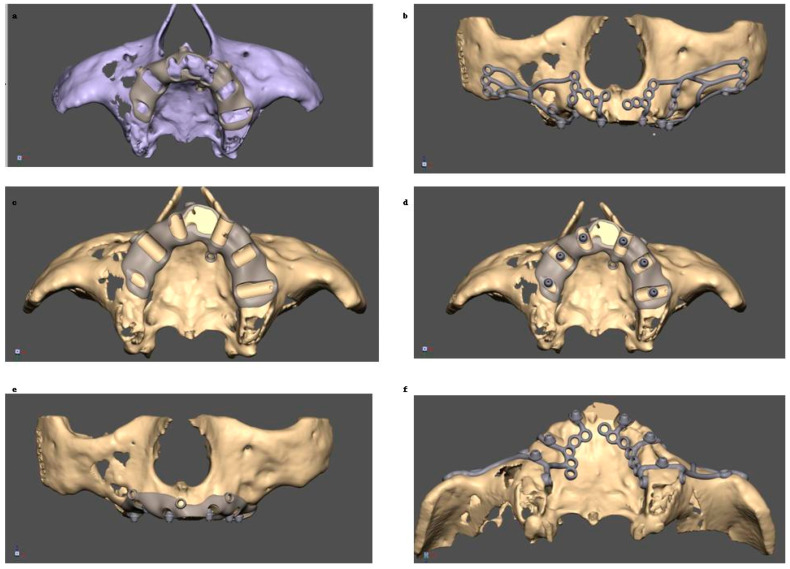
Process of designing a template to reduce bone on the alveolar ridge. (**a**) template put on alveolar ridge before bone reduction; (**b**,**f**) MaI Implant^®^ (Integra Implants^®^, Lodz, Poland) on the bone; (**c**) template put on alveolar ridge after bone reduction; (**d**,**e**) multiunits on reduced bone.

**Figure 3:**
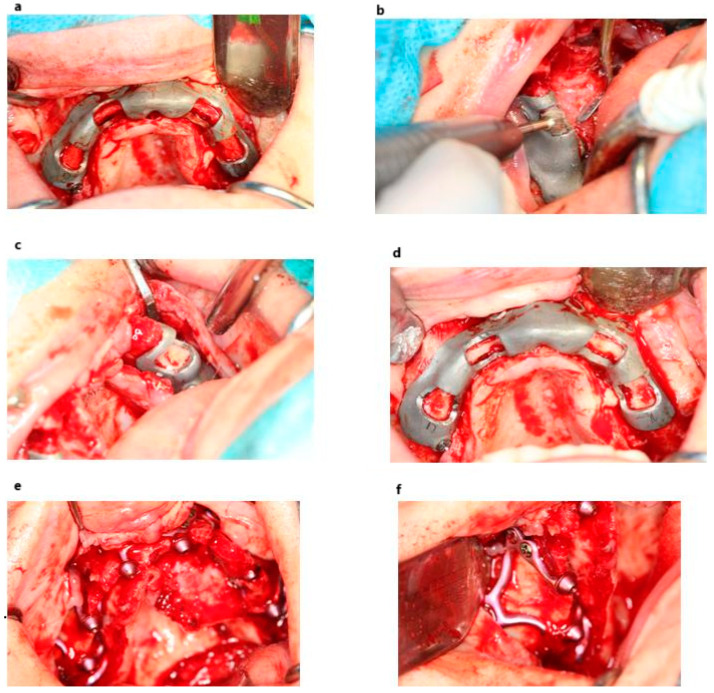
Clinical application of template (**a**–**d**) and the MaI Implant^®^ (Integra Implants^®^, Lodz, Poland) placement after bone adjustment (**e**,**f**).

**Figure 4:**
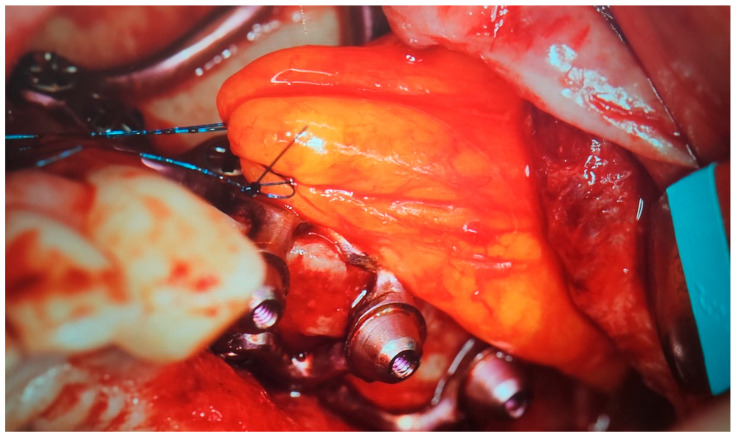
Bichat fat pad cover over the MaI Implant^®^ (Integra Implants^®^, Lodz, Poland). The fat pad can be sutured using resorbable sutures, in which case it is attached to the periosteum, or it can be sutured using non-resorbable sutures. However, in the latter case, the knot must be left above the gingiva so that it can be removed after a minimum of 10 days.

**Figure 5:**
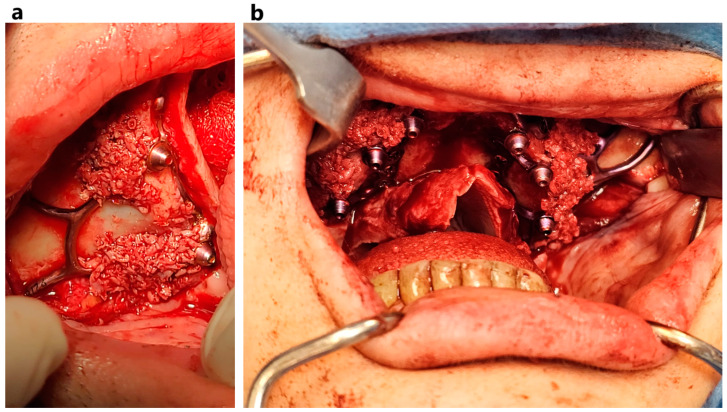
(**a**,**b**) bone chips were harvested using a disposable bone scraper and placed on the rami of the MaI Implant^®^ (Integra Implants^®^, Lodz, Poland). The aim of the bone chips was to reduce the risk of dehiscence of MaI Implant^®^.

Once adequate exposure was achieved, the custom-designed subperiosteal implant, manufactured to conform precisely to the patient’s maxillary anatomy, was positioned over the alveolar ridge (Figure 1). In a few cases, when the alveolar ridge was narrow and high, a template was used to reduce the bone (Figure 2 and Figure 3). When the Bichat fat pad fell out from its place during a surgical procedure, it was sutured on the vestibular surface of the MaI Implant^®^ (Integra Implants^®^, Lodz, Poland) (Figure 4). In a few cases, bone chips were harvested from the chin or external obliqua to cover the rami of the MaI Implant (Figure 5). The implant was secured using self-drilling screws with diameters of 2.0 mm or 2.2 mm, anodized in green or purple for differentiation. These screws were strategically placed at multiple anchorage points, including the zygomatic bone, palate, subnasal area, and mid-palatal suture. Screw lengths, ranging from 6 mm to 13 mm, were selected based on the density of the bone at each fixation site. Torque settings were adjusted accordingly, with values ranging from 5 Ncm for softer bone (Type IV) to 30 Ncm for denser bone (Type I), ensuring optimal stability.

In cases where soft tissue underlay was encountered or when the buccal fat pad was exposed during dissection, the fat pad was harvested and sutured to provide additional coverage and enhance implant integration. The surgical procedure was performed with precision, and the average duration of the surgery was approximately 174 min, with a standard deviation of ±43 min.

##### Postoperative Care

Postoperatively, temporary screw-retained acrylic bridges were delivered to all patients within 24 h, enabling immediate functional and esthetic restoration. For patients who underwent surgery under general anesthesia, a single dose of dexamethasone (8 mg) was administered intraoperatively to mitigate inflammation and postoperative swelling.

Patients were instructed to attend follow-up visits, during which sutures were removed two weeks after the surgery. Five months postoperatively, definitive prostheses were fabricated and delivered. These included permanent bridges made of materials such as acrylic fused to titanium, porcelain fused to CoCr, or fully contoured ZrO on titanium suprastructures, ensuring durable and high-quality restorations tailored to each patient’s needs.

#### 2.5.2. Zygomatic Implants

**Figure 6:**
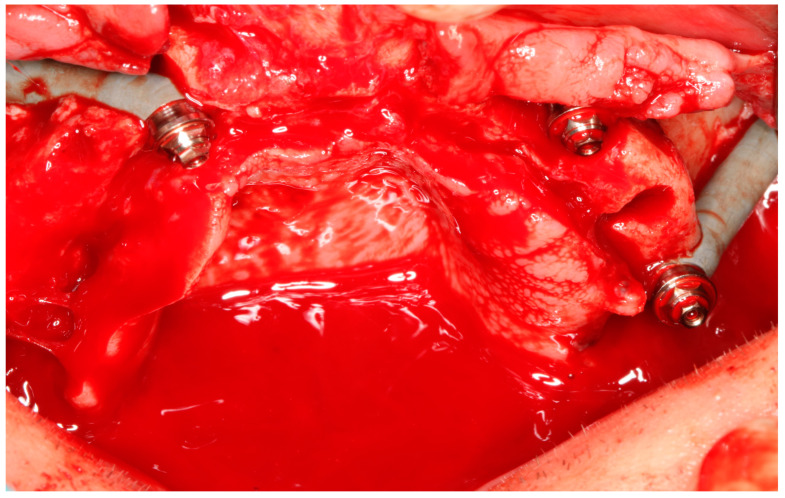
Extra-maxillary zygomatic implant placement.

The zygomatic implants were placed using a combination of the “sinus slot approach” and extra-sinus techniques, as needed, to achieve optimal implant anchorage and prosthetic support (Figure 6).

##### Preoperative Preparation

Prophylactic antibiotics (same regimen as for subperiosteal implants) were administered. All surgeries were performed under general anesthesia with nasal intubation to ensure patient comfort and surgical precision.

##### Surgical Technique

A crestal incision was made, extending from one maxillary tuberosity to the other, to provide full access to the surgical site. The palatal flap was carefully elevated, exposing the alveolar process and the infrazygomatic crest, which are critical anatomical landmarks for the procedure.

**Figure 7:**
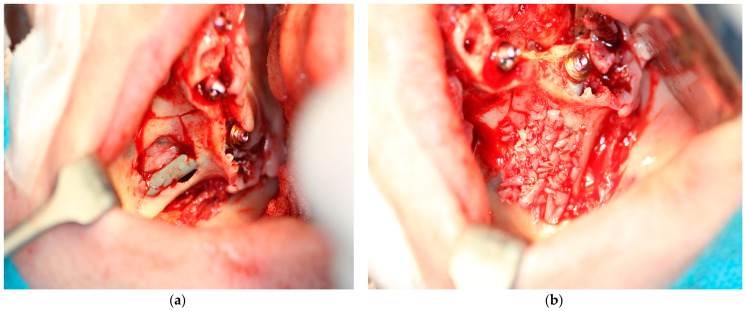
This picture shows the placement of a zygomatic implant using the Stella method (**a**) and bone chips harvested from the anterior maxillary sinus placed on the zygomatic implant in order to separate the implant from periosteal (**b**).

In some cases, bone chips were added on the surface of the zygomatic implants to enhance tissue coverage and optimize long-term integration. This approach, while not a standard requirement, was applied selectively based on clinical judgment and specific patient needs. A high-resolution photograph illustrating this technique has been included to provide further clarity (Figure 7).

To enable precise implant placement, a lateral sinus window was created using a diamond bur. This step allowed direct visualization of the sinus roof and facilitated safe and accurate positioning of the implants. The sinus mucosa was meticulously elevated to avoid perforation or damage, ensuring a clear path for implant insertion.

Drilling into the zygomatic bone was performed with an implant handpiece operating at a controlled speed of 600 rpm. Nobel Biocare zygomatic implants, featuring a TiUnite surface, 45-degree angulation, and a diameter of 4.3 mm, were then inserted. Implant lengths were chosen based on individual anatomical requirements, ranging from 30 mm to 52.5 mm.

The spatial relationship of each implant to the maxilla and sinus was carefully documented. Placement was categorized as intra- or extra-sinus and intramaxillary or extra-maxillary, depending on the implant’s position relative to these structures. This documentation ensured a detailed understanding of implant orientation and its integration within the surrounding anatomy.

##### Postoperative Care

Temporary screw-retained acrylic bridges were delivered within 24 h. Permanent prosthetic restorations were fabricated and delivered five months postoperatively. Patients underwent regular follow-up for five years, with emphasis on implant stability, sinus health, and prosthetic function.

## 3. Results

Some criteria have been taken into consideration during the comparison between subperiosteal implants and zygomatic implants (Table 2).

### 3.1. Implant Failure

**Figure 8:**
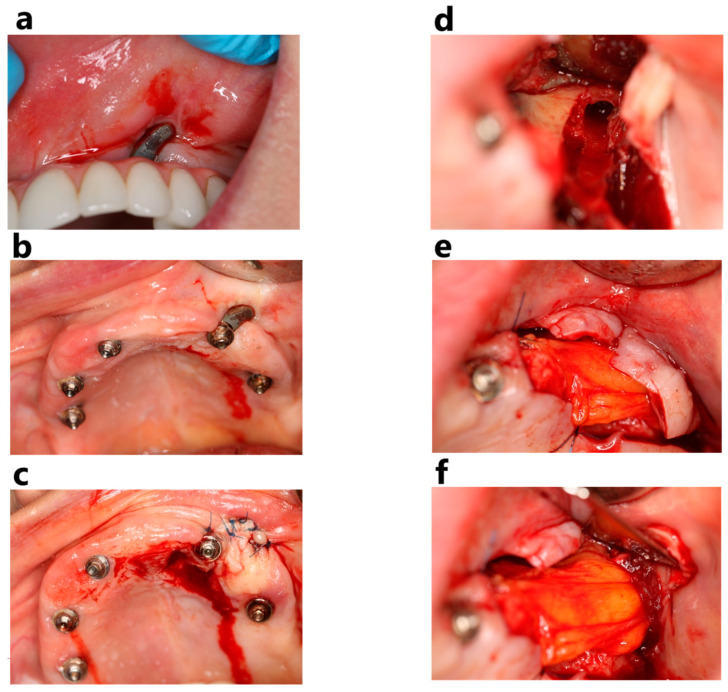
These pictures show soft tissue dehiscence around the zygomatic implant in the region of tooth 23 (**a**,**b**). An attempt was made to cover the exposed implant with a vascularized flap from the palate (**c**) after smoothing the implant with a bur. However, the gingiva became exposed again, and it was decided to remove the implant due to recurrent maxillary sinus infections. After unscrewing the implant (**d**), the cavity left by the removed implant was covered with Bichat’s fat pad (**e**,**f**).

**Figure 9:**
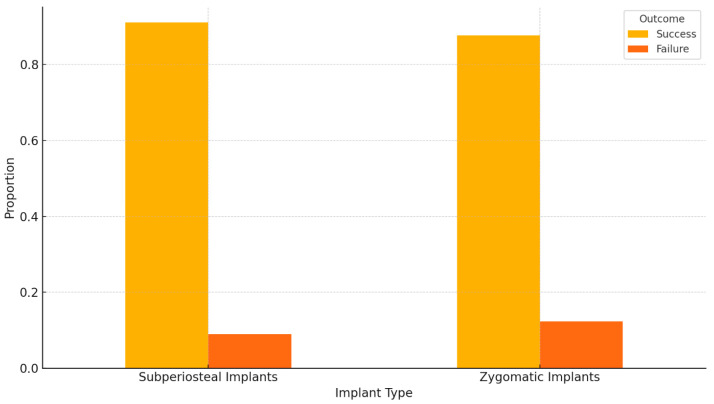
Bar chart comparing the success and failure rates of subperiosteal and zygomatic implants. *p*-value: **0.65** means there is no statistically significant difference in the success and failure rates between subperiosteal and zygomatic implants based on the data provided.

Individual implants demonstrated a lower failure rate compared to zygomatic implants, particularly in cases of severe maxillary atrophy (Figure 8). Although the survival rates of zygomatic (96.3%) and subperiosteal implants (97.1%) were not statistically significant (*p* = 0.278), the slightly higher survival rate of subperiosteal implants may be attributed to their customized design, which provides a precise fit to the patient’s anatomy. This individualized approach reduces stress concentrations on the implant and surrounding bone, enhancing stability and long-term integration (Figure 9).

### 3.2. Procedure Duration

**Figure 10:**
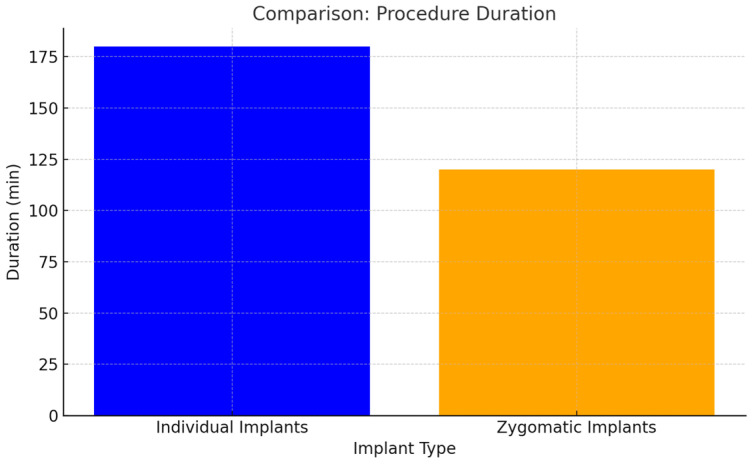
The longer procedure time for individual implants was due to the adjustments required to fit the bone to the template. Additionally, for subperiosteal implants, bone grafting from the lineaobliqua was performed, which further extended the duration of the procedure (*p* < 0.05).

While the placement of zygomatic implants required less surgical time, the meticulous placement of individual implants ensured greater precision and stability, leading to reduced long-term complications despite slightly longer procedures. Zygomatic implants were placed freehand without the use of a template, whereas some individual implants were placed with the guidance of a template. The longer procedure time for individual implants was due to the adjustments required to fit the bone to the template. Additionally, for subperiosteal implants, bone grafting from the lineaobliqua was performed, which further extended the duration of the procedure (Figure 10).

### 3.3. Maxillary Sinus Post-Surgical Lesions

**Figure 11:**
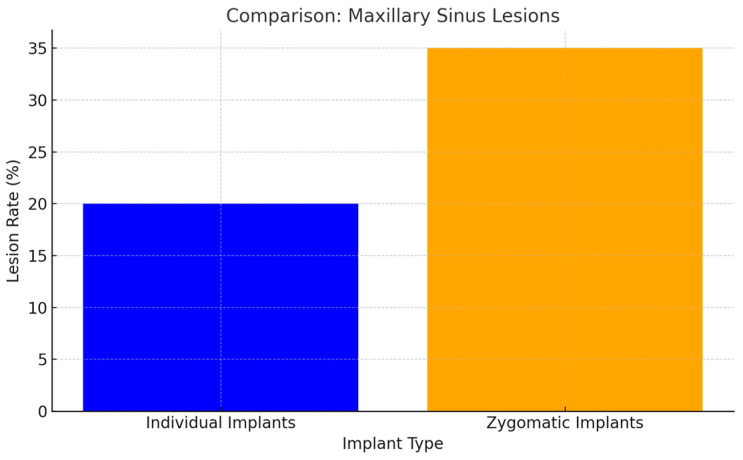
The design and placement technique of individual implants minimized the risk of sinus membrane perforation and associated complications (*p* < 0.05).

Patients with individual implants showed fewer post-surgical maxillary sinus lesions compared to those with zygomatic implants (*p* < 0.05). The design and placement technique of individual implants minimized the risk of sinus membrane perforation and associated complications (Figure 11).

### 3.4. Soft Tissue Recession

**Figure 12:**
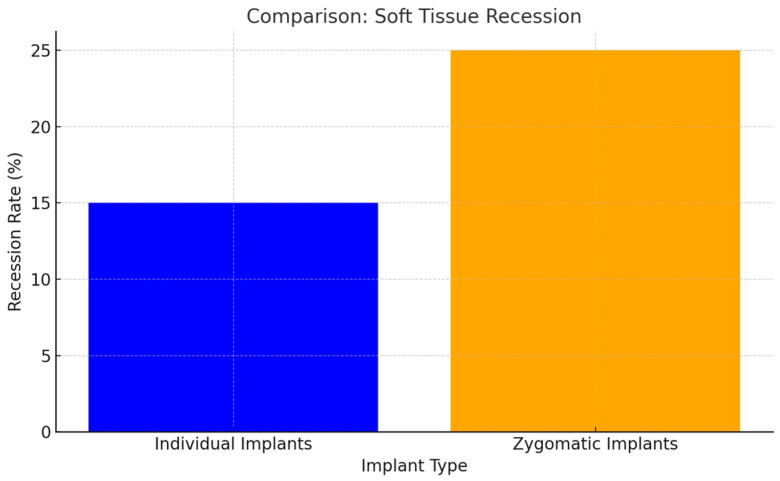
Extra-maxillary zygomatic implant placement carries a higher risk of soft tissue dehiscence (*p* < 0.05).

Soft tissue recession was significantly less frequent with individual implants than with zygomatic implants (*p* < 0.05). The precise design and positioning of individual implants contributed to better soft tissue integration and esthetic outcomes (Figure 12).

### 3.5. Membrane Thickness After Surgery

**Figure 13:**
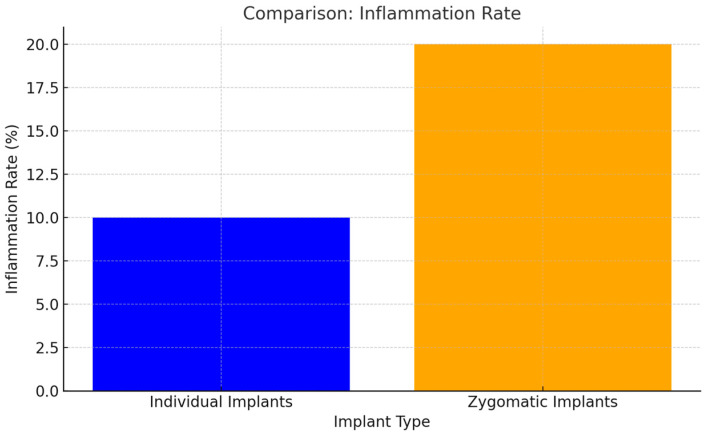
Sinusitis associated with extra-maxillary implant placement is a cause of inflammation observed both clinically around zygomatic implants and in CBCT imaging (*p* < 0.05).

Post-surgical membrane thickness was more stable in patients with individual implants. The reduced mechanical stress on surrounding structures associated with individual implants contributed to better postoperative outcomes (*p* < 0.05) (Figure 13).

### 3.6. Inflammation and Mobility

**Figure 14:**
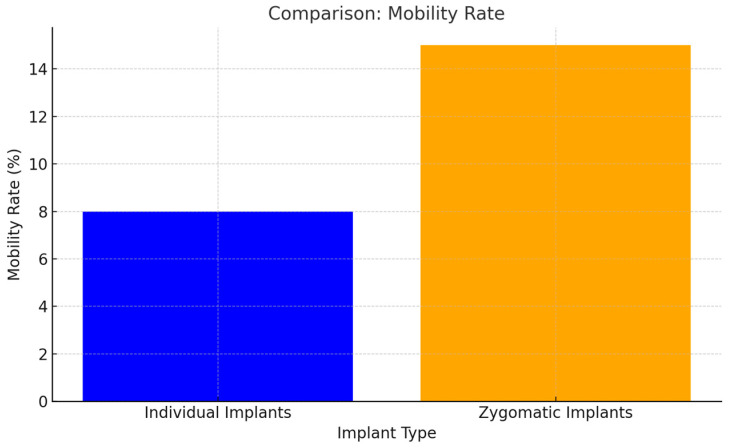
Anchorage in the zygomatic bone of zygomatic implants creates a cantilever effect. As a result, mobility at the distal end of zygomatic implants becomes apparent when pressure is applied with a dental instrument (*p* < 0.05).

Inflammation and implant mobility were notably lower in patients with individual implants compared to zygomatic implants. The enhanced stability and biocompatibility of individual implants were key factors in reducing these complications (*p* < 0.05) (Figure 14).

### 3.7. Statistical Analysis

Statistical analysis confirmed that the difference in procedure duration between the two groups was statistically significant (*p* < 0.05), as determined by the Kruskal–Wallis test. Similarly, trends observed for reduced complication rates, including lower implant failure, fewer maxillary sinus lesions, and less soft tissue recession in individual implants, were all statistically significant (*p* < 0.05) based on Chi-square tests. These results emphasize the clinical relevance of the observed differences in outcomes.

## 4. Discussion

The comparative evaluation of zygomatic and subperiosteal implants over a five-year follow-up highlights the clinical strengths and limitations of both modalities in addressing severe maxillary atrophy. This discussion synthesizes findings from our study and correlates them with evidence in the literature to provide a nuanced understanding of these advanced implant solutions. By expanding on the unique features and challenges of each approach, this section aims to provide a comprehensive perspective for clinical decision-making.

### 4.1. Implant Survival and Success Rates

Our findings demonstrate comparable survival rates between zygomatic and subperiosteal implants over five years, aligning with the high survival rates reported in the literature [11,12,13]. In our study, the survival rate for zygomatic implants was observed at 96.3%, while subperiosteal implants achieved a survival rate of 97.1%, with no statistically significant difference (*p* = 0.278). These findings reflect similar trends reported in the systematic reviews by Polido et al., 2023, Anitua et al., 2024, and Vaira et al., 2024, which documented survival rates exceeding 95% for both implant types [11,12,14].

Significant differences were noted in the nature of complications. Zygomatic implants showed higher incidences of sinus-related complications and risks of orbital damage [13,15]. Zygomatic implants showed a higher incidence of sinus-related complications (12.4%) compared to subperiosteal implants (5.6%), with a statistically significant difference (*p* < 0.05). Furthermore, subperiosteal implants demonstrated fewer incidences of soft tissue irritation and infection around fixation sites, which supports their role in anatomically complex cases [16]. The risk of orbital floor damage with zygomatic implants, although rare, remains a critical complication requiring advanced surgical planning and expertise, as highlighted in the literature [15].

Additionally, in cases of zygomatic implant failure due to a lack of osseointegration, alternative solutions are severely limited due to the resorption of the zygomatic bone post removal [13]. The zygomatic bone often undergoes significant resorption following the removal of a failed implant, leaving no possibility for re-implantation in the same region. This irreversible loss of bone contrasts sharply with the subperiosteal approach. In cases of subperiosteal implant failure, clinicians can allow a three-month healing period before attempting re-implantation. This reattempt, facilitated by modifying the implant’s design to adapt to the healed anatomy, provides a flexible and reliable alternative that is not feasible with zygomatic implants.

In this study, the survival rate of zygomatic implants and subperiosteal implants was similar, with a slight advantage for subperiosteal implants. A possible reason for this could be that zygomatic implants, while effective for immediate functional loading, rely on standardized designs that may not account for individual anatomical variations. Additionally, their placement often involves anchorage in the zygomatic bone, a site susceptible to mechanical stress due to cantilever forces, which could potentially increase the risk of minor complications affecting implant survival in the long term.

Other factors, such as operator experience, patient selection criteria, and variations in postoperative care, may also contribute to the observed differences. Further studies with larger cohorts and detailed subgroup analyses are warranted to explore these factors in greater depth.

### 4.2. Functional and Prosthetic Outcomes

Functionality and prosthetic success are critical metrics in assessing implant effectiveness. Both zygomatic and subperiosteal implants enable immediate loading, as has been confirmed in our research. Both zygomatic and subperiosteal implants demonstrated the capacity for immediate loading, ensuring the prompt restoration of function and esthetics for patients, which aligns with modern demands for shorter treatment timelines and enhanced patient satisfaction [17]. The ability to immediately load zygomatic implants reflects their robust anchorage in the zygomatic bone and shorter treatment times.

Subperiosteal implants, however, provided superior long-term soft tissue stability, with fewer cases of peri-implantitis. In this research the critical importance of keratinized mucosa for maintaining peri-implant soft tissue health has been emphasized.

### 4.3. Patient-Specific Considerations

The choice between zygomatic and subperiosteal implants often depends on patient-specific factors, including anatomical and systemic considerations. Subperiosteal implants’ adaptability to individual anatomical variations makes them preferable in cases with contraindications for zygomatic implant placement due to sinus or orbital proximity [9].

Additionally, zygomatic implants demonstrated significant advantages in cases requiring immediate restoration, especially when immediate implantation is performed. However, their proximity to critical anatomical structures such as the orbit and maxillary sinus demands a high level of surgical expertise to mitigate complications [2]. Subperiosteal implants, with their ability to adapt to individual anatomical variations, provide a safer alternative for patients with complex anatomical challenges and contraindications to invasive approaches [18].

### 4.4. Cost Considerations

A critical consideration in evaluating zygomatic and subperiosteal implants is the cost of manufacturing. Subperiosteal implants involve advanced imaging and 3D printing workflows, which contribute to higher costs relative to mass-produced zygomatic implants [9]. Subperiosteal implants are custom designed to fit the unique anatomical structure of each patient, requiring advanced imaging, computer-aided design (CAD), and additive manufacturing techniques. These processes contribute to higher production costs compared to standardized zygomatic implants. Zygomatic implants, typically mass-produced with standardized designs, benefit from economies of scale, potentially reducing manufacturing expenses [19]. However, the overall cost-effectiveness of each implant type depends not only on production costs but also on factors such as surgical complexity, clinician expertise, and long-term outcomes, including complication rates. These economic considerations are integral to clinical decision-making, especially in resource-limited settings.

### 4.5. Strategies to Reduce Surgical Risks

Advanced preoperative imaging, such as cone beam computed tomography (CBCT), plays a critical role in surgical planning by providing high-resolution, three-dimensional anatomical details. These images allow for the precise mapping of critical structures, such as the maxillary sinus and orbital floor, minimizing the risk of perforation or damage during implant placement [20].

Guided surgery techniques, utilizing digital workflows and surgical templates, further enhance accuracy by translating virtual plans into precise intraoperative execution. These technologies reduce variability in implant angulation and depth, ensuring optimal placement even in complex anatomical scenarios [21].

Intraoperative navigation systems, which provide real-time feedback during surgery, offer additional safeguards by allowing surgeons to adjust implant placement dynamically. This is particularly beneficial in cases of severe maxillary atrophy, where small deviations can result in significant complications.

The integration of these advanced tools not only reduces surgical risks but also shortens learning curves for less experienced surgeons, thereby improving overall patient outcomes. Future innovations, such as augmented reality-assisted surgery, hold promise for further enhancing the safety and precision of implant procedures [22].

### 4.6. Clinical Implications and Future Directions

The results of our study underscore the importance of individualized treatment planning in selecting the appropriate implant modality. While zygomatic implants excel in immediate functional loading and shorter treatment times, their associated risks, particularly orbital floor damage and sinus complications, require advanced surgical skills and careful patient selection. Subperiosteal implants, on the other hand, excel in providing long-term stability and fewer complications, particularly in anatomically complex cases. The integration of advanced digital workflows and additive manufacturing has significantly enhanced the safety and predictability of subperiosteal implants, as demonstrated in our study.

Future research should focus on long-term follow-ups and randomized controlled trials comparing these modalities to strengthen evidence-based recommendations. Additionally, innovations in biomaterials and regenerative techniques to enhance keratinized mucosa and peri-implant tissue integration may further optimize outcomes. Exploring patient-reported outcomes such as quality of life and satisfaction will provide a more holistic understanding of the benefits of each implant modality.

## 5. Conclusions

In conclusion, zygomatic and subperiosteal implants represent complementary solutions for managing severe maxillary atrophy. Their respective advantages highlight the critical role of individualized treatment planning. Subperiosteal implants, with their customized design and lower risk of anatomical complications, may offer a safer alternative in specific clinical scenarios, while zygomatic implants remain a robust option for immediate loading in experienced hands. The presence of keratinized mucosa emerges as a key factor in ensuring the long-term success of subperiosteal implants. Further advancements in technology and technique will continue to shape the future of implant dentistry, optimizing outcomes for a diverse patient population. Moreover, the continuous evolution of surgical methods and digital planning technologies promises to expand the indications and improve the predictability of both zygomatic and subperiosteal implants, paving the way for even more reliable solutions in managing severe maxillary atrophy.

## Figures and Tables

**Table 1:** Comparison of patient groups for subperiosteal and zygomatic implants.

Group	Configuration
Subperiosteal Implants Group 1	Two individual subperiosteal implants, each with three multiunits
Subperiosteal Implants Group 2	Two individual subperiosteal implants combined with one or two conventional implants, each with two multiunits
Subperiosteal Implants Group 3	A single subperiosteal implant with two, three, or four multiunits
Zygomatic Implants Group 1	Four zygomatic implants combined with one or two conventional implants
Zygomatic Implants Group 2	Four zygomatic implants exclusively
Zygomatic Implants Group 3	Two zygomatic implants combined with four conventional implants
Zygomatic Implants Group 4	Three zygomatic implants combined with one or two conventional implants

**Table 2:** Comparison of zygomatic implants vs. MaI^®^ Implants.

Criteria	Zygomatic Implants	Subperiosteal Implants
Implant Survival Rate	96.3%	97.1%
Complication Rate	Higher incidence of sinus-related complications and orbital damage (12.4%)	Lower incidence of soft tissue complications (5.6%)
Immediate Loading	Enabled	Enabled
Procedure Duration	Shorter	Longer (harvesting bone chips, suturing Bichat fat pad)
Soft Tissue Stability	Lower	Higher
Maxillary Sinus Lesions	Higher	Lower
Cost Considerations	Lower (mass-produced, standardized designs)	Higher (customdesigned, 3D printed)

## Data Availability

Data are unavailable due to privacy or ethical restrictions.
